# Simulation of Wheat Response to Future Climate Change Based on Coupled Model Inter-Comparison Project Phase 6 Multi-Model Ensemble Projections in the North China Plain

**DOI:** 10.3389/fpls.2022.829580

**Published:** 2022-02-03

**Authors:** Huizi Bai, Dengpan Xiao, Bin Wang, De Li Liu, Jianzhao Tang

**Affiliations:** ^1^Engineering Technology Research Center, Geographic Information Development and Application of Hebei, Institute of Geographical Sciences, Hebei Academy of Sciences, Shijiazhuang, China; ^2^College of Geography Science, Hebei Normal University, Shijiazhuang, China; ^3^Hebei Laboratory of Environmental Evolution and Ecological Construction, Shijiazhuang, China; ^4^NSW Department of Primary Industries, Wagga Wagga Agricultural Institute, Wagga Wagga, NSW, Australia; ^5^Climate Change Research Centre and ARC Centre of Excellence for Climate Extremes, University of New South Wales, Sydney, NSW, Australia

**Keywords:** climate change, heat stress, frost stress, CMIP6, wheat yield, APSIM

## Abstract

Global climate change results in more extreme temperature events, which poses a serious threat to wheat production in the North China Plain (NCP). Assessing the potential impact of temperature extremes on crop growth and yield is an important prerequisite for exploring crop adaptation measures to deal with changing climate. In this study, we evaluated the effects of heat and frost stress during wheat sensitive period on grain yield at four representative sites over the NCP using Agricultural Production System Simulator (APSIM)-wheat model driven by the climate projections from 20 Global Climate Models (GCMs) in the Coupled Model Inter-comparison Project phase 6 (CMIP6) during two future periods of 2031–2060 (2040S) and 2071–2100 (2080S) under societal development pathway (SSP) 245 and SSP585 scenarios. We found that extreme temperature stress had significantly negative impacts on wheat yield. However, increased rainfall and the elevated atmospheric CO_2_ concentration could partly compensate for the yield loss caused by extreme temperature events. Under future climate scenarios, the risk of exposure to heat stress around flowering had no great change but frost risk in spring increased slightly mainly due to warming climate accelerating wheat development and advancing the flowering time to a cooler period of growing season. Wheat yield loss caused by heat and frost stress increased by −0.6 to 4.2 and 1.9–12.8% under SSP585_2080S, respectively. We also found that late sowing and selecting cultivars with a long vegetative growth phase (VGP) could significantly compensate for the negative impact of extreme temperature on wheat yields in the south of NCP. However, selecting heat resistant cultivars in the north NCP and both heat and frost resistant cultivars in the central NCP may be a more effective way to alleviate the negative effect of extreme temperature on wheat yields. Our findings showed that not only heat risk should be concerned under climate warming, but also frost risk should not be ignored.

## Introduction

Climate change, as one of the most important factors that determine crop yield, could explain 30–50% of global yield variability (Ray et al., 2015; Rezaei et al., 2018). Along with warming climate, the intensity, frequency, and duration of extreme climate events are also increasing, which can exacerbate the instability of agricultural production systems (Zheng et al., 2012; Chen et al., 2018). Predicting the potential impact of future climate change and climate extreme on agricultural production is crucial for developing adaptation strategies to reduce climate risks (Chen et al., 2018).

China is currently the largest wheat-producing country in the world, accounting for more than 17.6% of the global wheat production (FAO, 2013). The North China Plain (NCP) is one of the major winter wheat planting areas in China, accounting for over 50% of China’s total wheat production (Xiao et al., 2020). Therefore, ensuring wheat production in the NCP is not only for food security in China but also for the stability and sustainability of the global food market. However, wheat production will be threatened by the increased extreme climate events. For example, short episodes of heat stress during flowering can lead to sterility and abortion of grains, resulting in a low grain number (Ferris et al., 1998). During the grain filling period, heat stress can accelerate leaf senescence and affect final grain weight by shortening the duration of grain filling (Zhao et al., 2007; Dias and Lidon, 2009). For every unit increase of the sum of daily heat degrees over 30°C during anthesis and grain filling, grain yield was reduced by 1.0–1.6% (Liu et al., 2016).

Along with climate warming, most previous studies have been well concerned with the effect of heat stress on crop production (Talukder et al., 2014; Liu et al., 2016; Chen et al., 2018). However, the advancement of crop phenological stages caused by increasing temperature has the potential to increase the risk of spring frost (Saeidi et al., 2012; Chen et al., 2014). Actual frost risk for crop does not decrease as expected and shows an increasing trend in the future in some regions (He et al., 2012; Crimp, 2014). Moreover, experiencing a warm winter for winter wheat is more vulnerable to low temperature stress in the spring because warm winter could affect the process of cold hardening and even cause dehardening (Bokhorst et al., 2010; Li et al., 2015a). In the NCP, frost injury usually occurs in March and April during jointing and booting stages (Wu et al., 2014; Li et al., 2015a). Generally, the frost stress-induced yield loss is attributed mainly to the reduction of tiller and spike number, which are associated with a decrease in photosynthetic rate, specific leaf area, and shoot biomass (Valluru et al., 2012; Thakur and Nayyar, 2013; Li et al., 2015b). For example, each additional day in low temperature duration at jointing and booting stages could reduce grain yield by 4.3–4.8 and 5.2–6.7% for two different wheat cultivars under the minimum temperature of −2°C, respectively (Ji et al., 2017).

Generally, proper adjustment of cultivars and sowing dates can reduce the impact of extreme climate stress on crop production (Bai et al., 2020). Gouache et al. (2012) showed that compared to the reference strategy, earlier sowing wheat by 30 days can decrease heat stress days by 0.9 days, and heat-tolerant cultivar can reduce heat stress days by 3.5 days during wheat grain filling in the future period 2070–2099. In addition, late sowing date and using long-season cultivars could be possible strategies to minimize frost damage for wheat (Zheng et al., 2012; Xiao et al., 2018a).

Process-based crop models are valuable tools for evaluating the impact of climate change on crop production (Challinor et al., 2014). Numerous studies have employed crop models to assess the effects of temperature changes on crop production (Lobell and Asner, 2003; Tao and Zhang, 2013; Asseng et al., 2015). However, these studies mainly consider the impact of mean temperature on crop development and yield and ignore the effect of extreme temperature on crop production, which could underestimate climate change impacts on crop yield (Tao et al., 2012; Wang et al., 2013). This is because most crop models cannot directly and effectively simulate the effects of heat or frost stress on crop development, growth, and yield (Xiao and Tao, 2014). Currently, several studies have tried to consider extreme climate stress by incorporating a series of stress functions that accelerate leaf senescence and reduce grain number and yield into present crop models (Stöckle et al., 2003; Barlow et al., 2015; Bell et al., 2015). Using these improved crop models, some studies evaluated the response and adaptation of crop to extreme temperature under future climate scenarios, but most studies only focused on a type of extreme temperature, that is, heat stress (Gouache et al., 2012; He et al., 2012; Chen et al., 2018). To more realistically assess crop yields and explore adaptation measures for future climate change, it is necessary to analyze the potential impact of the co-occurrence of frost and heat stress on crop yield.

In this study, we aim to assess the impacts of future climate change mainly including heat and frost stress on wheat yield in the NCP using the Agricultural Production System Simulator (APSIM) model forced by statistically downscaled daily climate data from 20 Global Climate Models (GCMs) in the Coupled Model Inter-comparison Project phase 6 (CMIP6). The objectives of this study were to (1) investigate the response of wheat phenology and yield to future climate change in the NCP; (2) separately and jointly evaluate the impacts of heat and frost stress on wheat production; and (3) identify the optimum cultivar and sowing time to minimize the risk of yield loss mainly caused by heat and frost stress under future climate scenarios.

## Materials and Methods

### Study Sites

**Figure 1:**
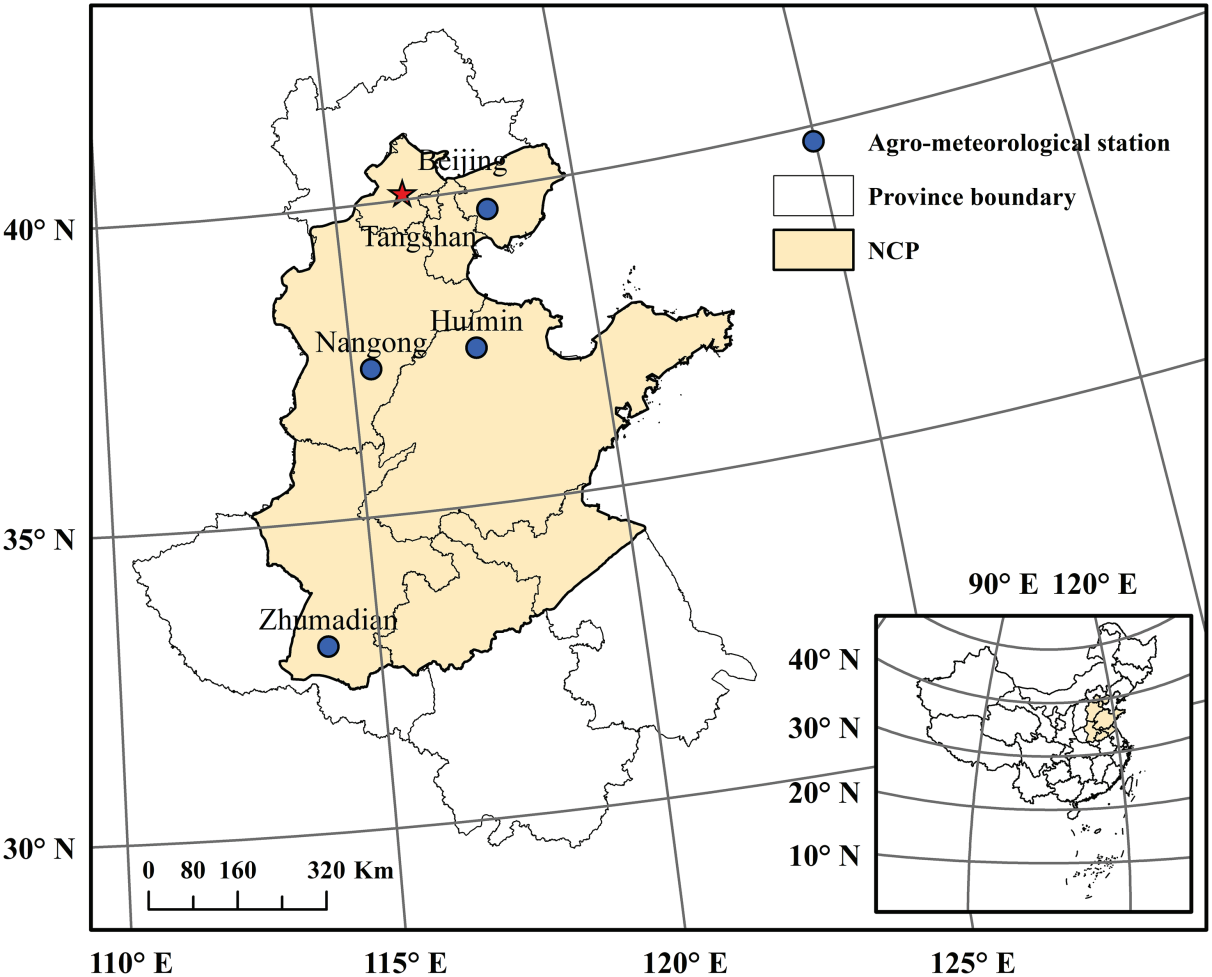
Locations of the four investigated agro-meteorological stations in the North China Plain (NCP).

The NCP as shown in Figure 1 is an important base for wheat production in China. The main soil type in the NCP is loam of Aeolian origin, a soil type deposited by rivers over geological periods. The NCP has a warm and semi-humid continental monsoon climate. Seasonal precipitation is not evenly distributed, 50–80% of which occurs in the period from July to September. The NCP has a reliance on irrigation to support the intensive double cropping system with rotation between winter wheat and summer maize. Winter wheat is grown from early October to June of the following year (the average growth period in the past 30 years) under irrigated conditions and intensive use of fertilizer, pesticides, and herbicides (Xiao et al., 2013; Xiao and Tao, 2014). After fully considering the completeness and quality of crop and climate data, we selected four representative sites in the northern, central, and southern parts of the North China Plain respectively, including Tangshan (TS) and Nangong (NG) stations in Hebei province, Huimin (HM) station in Shandong province and Zhumadian (ZMD) station in Henan province (Figure 1). All the selected sites are typical winter wheat production zones, representative of typical double cropping system in the NCP, which are geographically and climatologically different and have good records on weather/crop data. Detailed information about crop and climate for these four stations was given in Table 1.

**Table 1:** General information about crop and climate data for the four investigated stations in the North China Plain (NCP).

Station	Tangshan (TS)	Nangong (NG)	Huimin (HM)	Zhumadian (ZMD)
**Crop data**				
Year	2005–2008	2006–2008	2005–2009	2006–2009
Cultivar	Jingdong8	Shimai12	Lumai23	Zhengmai9023
Mean sowing date (DOY)	277	285	281	295
Mean jointing date (DOY)	111	94	98	70
Mean flowering date (DOY)	132	122	126	104
Mean maturity date (DOY)	166	157	159	139
Mean yield (kg ha^−1^)	6415.4	6442.6	6833.2	5373.0
**Climate data**				
Mean daily temperature (°C)	12.8	14.0	13.5	15.7
Mean daily solar radiation (MJ m^−2^)	13.9	13.3	13.6	12.1
Total precipitation (mm)	587.7	454.4	538.9	999.7
Soil type	Loam	Loam	Loam	Silty loam

### Climate and Crop Data

Crop data for wheat at the four stations, including crop phenological stages, yield, and agronomic management practices, were also obtained from the CMA. Crop management practices at the stations were generally same as the local farmer’s practices (Tao et al., 2014). Winter wheat at ZMD (high-rainfall site) was maintained under rainfed, while regular irrigation was applied to winter wheat at the other three stations. About 100 and 60 kg/ha nitrogen were applied at sowing and jointing, respectively.

Historical daily climate data from 1961 to 2014 at the four selected stations were obtained from the Chinese Meteorological Administration (CMA), including minimum (T_min_) and maximum temperature (T_max_), sunshine hours, and precipitation (Prec). Daily solar radiation (Rad) was calculated from daily sunshine hours using the Angstrom equation (Prescott, 1940).

Future climate projections were based on 20 GCMs from CMIP6 (Table 2).^1^ These climate projections were driven by a new set of integrated assessment models (IAMs) based on the Shared Socioeconomic Pathways (SSPs) and the Representative Concentration Pathways (RCPs; O'Neill et al., 2016). In this study, we used future climate projections for two integrated scenarios (combining SSP2 with RCP4.5, defined by SSP245 and combining SSP5 with RCP8.5, defined by SSP585). SSP2 envisions a central pathway in which social, economic, and technological trends do not shift markedly from historical patterns. SSP5 envisions fossil-fueled development pathway with rapid technological progress and development of human capital (O'Neill et al., 2016). RCP4.5 is a medium radiative forcing pathway (4.5 W m^−2^ in 2100), and RCP8.5 is a high radiative forcing pathway (8.5 W m^−2^ in 2100).

**Table 2:** List of the 20 Global Climate Models (GCMs) for future climate projections used in this study.

Code	GCM name	Abbreviation	Institute ID	Country	Spatial resolution of atmospheric model
1	ACCESS-CM2	ACC1	CSIRO–ARCCSS	Australia	1.9° × 1.3°
2	ACCESS-ESM1-5	ACC2	CSIRO–ARCCSS	Australia	1.9° × 1.3°
3	BCC-CSM2-MR	BCC	BCC	China	1.1° × 1.1°
4	CanESM5	CAN1	CCCMA	Canada	2.8° × 2.8°
5	CanESM5-CanOE	CAN2	CCCMA	Canada	2.8° × 2.8°
6	CNRM-CM6-1	CNR1	CNRM	France	1.4° × 1.4°
7	CNRM-ESM2-1	CNR2	CNRM	France	1.4° × 1.4°
8	EC-Earth3-Veg	ECE1	EC–EARTH	Europe	0.7° × 0.7°
9	EC-Earth3	ECE2	EC–EARTH	Europe	0.7° × 0.7°
10	FGOALS-g3	FGO	FGOALS	China	2.0° × 2.3°
11	GFDL-ESM4	GFD	NOAA–GFDL	America	1.0° × 1.0°
12	GISS-E2-1-G	GIS	NASA–GISS	America	2.5° × 2.0°
13	INM-CM4-8	INM1	INM	Russia	2.0° × 1.5°
14	INM-CM5-0	INM2	INM	Russia	2.0° × 1.5°
15	IPSL-CM6A-LR	IPS	IPSL	France	2.5° × 1.3°
16	MPI-ESM1-2-HR	MPI1	MPI-M	Germany	0.9° × 0.9°
17	MPI-ESM1-2-LR	MPI2	MPI-M	Germany	1.9° × 1.9°
18	MIROC6	MIR1	MIROC	Japan	1.4° × 1.4°
19	MIROC-ES2L	MIR2	MIROC	Japan	2.8° × 2.8°
20	MRI-ESM2-0	MRI	MRI	Japan	1.9° × 1.9°

Since the spatial resolution of different GCMs varied greatly and the crop data was site-based, we uniformly downscaled the grid data to sites. In addition, the APSIM model is driven by daily climate data. In this study, the monthly gridded data projected by the GCMs were downscaled to daily climate series for the four selected stations using a statistical downscaling method developed by Liu and Zuo (2012). This approach used monthly gridded GCM climate data and parameters derived from GCM projections and climate observations to generate a realistic time series of daily temperature, precipitation, and solar radiation. Firstly, monthly GCM simulations of the different climate variables were downscaled to specific stations using the inverse distance-weighted interpolation method (IDW). Secondly, quantile-quantile bias correction approach is applied to ensure that the model-derived monthly data matches well with the observed data for a historical training period (Liu and Zuo, 2012). Finally, daily climate data for each station were generated from the spatially downscaled monthly GCM projections using the modified stochastic weather generator (WGEN; Richardson and Wright, 1984). More detailed description of the method can be found in Liu and Zuo (2012). Bai et al. (2020) assessed the performance of the downscaled GCMs data from CMIP6 in reproducing historical changes of extreme climate indices using the multi-model arithmetic mean and found that the ensemble results could better reproduce historical changes of extreme climate than any individual GCM. In this study, we downscaled climate inputs for APSIM model for the 1961–2100 period at the four agro-meteorological stations under each of the 20 GCMs for SSP245 and SSP585.

### APSIM-Wheat Model and Setting for Frost and Heat Stress

Agricultural Production System Simulator model is a biophysical model to simulate crop growth and development on a daily time step (Holzworth et al., 2014). It can be used to mimic the response of single crop or crop rotations to climate change with different management practices (Holzworth et al., 2014). In APSIM-wheat model, wheat phenological development is defined by 11 crop stages and 10 crop phases (time between stages). The time of each phase is mainly determined by the accumulation of thermal time adjusted for other factors (e.g., vernalization and photoperiod) which vary with the phase considered. A more detailed description of the model is documented at http://www.apsim.info. In our study, APSIM-wheat version 7.10 was used to evaluate the responses of winter wheat yield to climate change, cultivar and sowing date adjusting.

In the NCP, wheat growth and development often suffer from heat and frost stress events. However, the APSIM-wheat model does not consider the effects of heat and frost stress on wheat yield (Bell et al., 2015). Under the background of global warming, it is very important to assess the impacts of heat and frost stress on wheat production. In the study area, frosts usually occur 3 or 4 weeks before head emergence when wheat is at the jointing stage (Zhong et al., 2008). In addition, cold stress during the period from booting to flowering can reduce the grain number per spike and decreased grain yield (Subedi et al., 1998). Based on previous studies and field trial data, spring frost damage generally occur when daily minimum temperature is below 0°C (Barlow et al., 2015; Zheng et al., 2015). Moreover, related studies indicated that wheat is more sensitive to heat stress that occurs at anthesis than it occurs during grain filling (Kang, 2015; Liu et al., 2016). The optimum temperature for wheat flowering and grain filling ranges from 19 to 22°C (Porter and Gawith, 1999). The threshold of 32°C is commonly regarded as the upper base temperature during the period of pre-anthesis (Porter and Gawith, 1999; Yang et al., 2017). For the grain filling period, the upper base temperature is between 33.4 and 37.4°C (Russell and Wilson, 1994; Porter and Gawith, 1999). We used the threshold of 35°C as the upper base temperature during the grain filling period in the NCP (Liu et al., 2016; Yang et al., 2017).

In this study, yield loss was calculated by multiplying influence coefficients depending on the frequency and intensity of heat and frost events during the critical phenological phase (Table 3). Since there are not sufficient data to test the impacts of frost or heat stress on winter yields for different cultivars at each site, yield reduction multipliers for heat and frost stress events are all the same in APSIM model for different sites based on the study of Bell et al. (2015). Although this approach in APSIM has not been fully calibrated, it provides some helpful information to capture the loss of crop yield due to heat and frost stress (Bell et al., 2015). This approach has been widely used to explore optimum flowering periods for wheat in Australia (Luo et al., 2018; Chen et al., 2020).

**Table 3:** Temperature criteria and yield reductions caused by frost and heat stress during wheat growth stages (Zadoks growth stage).

Stress	Sensitive stage (corresponding phenological phase)	Temperatures condition for frost or heat stress	Yield loss (% per day)
Frost	Z31–60^a^ (jointing to flowering)	T_min_ ≤ −2°C	5%
	Z60–79 (flowering to grain filling)	T_min_ ≤ 0°C	10%
Heat	Z55–65 (pre-flowering)	T_max_ ≥ 32°C	10%
	Z65–79 (post-flowering to grain filling)	T_max_ ≥ 35°C	10%

a Note that Zadoks growth stage from Zadoks et al. (1974).

### Simulation Setting

The APSIM-wheat model at four investigated stations was calibrated and validated in Xiao et al. (2018b). The model was robust to simulate dates of flowering and maturity with the root-mean-square deviation (RMSD) less than 5 days and yield with RMSD less than 10% compared to the observed value (Supplementary Figure S1). Detailed information of field management practices (e.g., sowing density, fertilization, and irrigation) referred to Xiao et al. (2018b). Based on long-term historical phenology records, sowing dates of wheat at four investigated stations were shown in Table 1. In this study, we mainly focused on the analyses of the impact of climate change (including heat and frost stress), cultivar, and sowing dates on wheat yield for three different 30-year periods under SSP245 and SSP585 scenarios, including the historical period of 1981–2010 (referred to as the baseline period) and the two future periods, that is, 2031–2060 (referred to as the 2040S) and 2071–2100 (referred to as the 2080S).

Elevated atmosphere CO_2_ concentration [(CO_2_)] in the future can significantly affect crop yield. During the baseline period, we set the [CO_2_] to 380 ppm. The yearly atmospheric [CO_2_] for the two future periods were calculated using empirical equations that were obtained by non-linear least-squares regression, based on the concentration pathway given by The Scenario Model Inter-comparison Project (ScenarioMIP) for CMIP6 (O'Neill et al., 2016). The empirical equations for calculating [CO_2_] for SSP245 and SSP585 are as follows:


(1) $$\begin{array}{c} CO_{2,SSP245}=62.044+\frac{34.002-3.8702y}{0.24423-1.1542y^{2.4901}}\\ \;+0.028057{\left(y-1900\right)}^{2}+0.00026827{\left(y-1960\right)}^{3}\\ \;-9.2751\times 10^{-7}{\left(y-1910\right)}^{4}-2.2448\left(y-2030\right) \end{array}$$

(2) $$\begin{array}{c} CO_{2,SSP585}=757.44+\frac{84.938-1.537y}{2.2011-3.8289y^{-0.45242}}\\ \;+2.4712\times 10^{-4}{\left(y+15\right)}^{2}+1.9299\times 10^{-5}{\left(y-1937\right)}^{3}\\ \;+5.1137\times 10^{-7}{\left(y-1910\right)}^{4} \end{array}$$

where *y* is the calendar year from 1900 to 2100 (i.e., *y* = 1900, 1901, …, 2100).

### Evaluating the Impacts of Heat and Frost Stress on Wheat Production

In this study, we evaluated the impacts of heat and frost stress on wheat yield at four study sites in the NCP with and without changing sowing time and cultivar. Impact of extreme stress on yield in the baseline period ( $Y_{bc}$ ) at each station for each GCM was identified as


(3) $$Y_{bc}=\frac{Y_{B}-Y_{A}}{Y_{A}}\times 100\%$$

where $Y_{A}$ and $Y_{B}$ were annual average of simulation results for baseline period without and with the effect of extreme stress, respectively.

The relative change of impacts of extreme stress on crop yield in the future period ( $Y_{\mathrm{fc}}$ ) compared to baseline at each station for each GCM was defined as:


(4) $$Y_{\mathrm{fc}}=\left(\frac{Y_{D}-Y_{C}}{Y_{A}}-Y_{bc}\right)\times 100\%$$

where $Y_{C}$ and $Y_{D}$ were average simulated yield for the future period without and with the effect of extreme stress, respectively.

### Optimizing Cultivar and Sowing Time Under Future Climate Scenarios

Using agronomic adaptation options is an effective option to improve yield performance (Bai and Tao, 2017). In this study, we investigated the wheat yield performance of historical cultivar (HC) and three virtual cultivars [thermal time in the vegetative growth phase (VGP) of HC increased by 10%, VC1; thermal time in the reproductive growth phase (RGP) of HC increased by 10%, VC2; thermal time in both the vegetative and RGPs of HC increased by 10%, VC3] for each station under future climate scenarios without and with the effect of extreme stress. Phenological parameters for HCs and three created wheat cultivars in the APSIM-Wheat model were shown in Table 4.

**Table 4:** Phenological parameters for historical cultivar (HC) and three virtual wheat cultivars (VC1, VC2, and VC3) in the Agricultural Production System Simulator (APSIM)-wheat model.

Station	Cultivar	tt_end_of_juvenile	tt_floral_initiation	startgf_to_mat	vern_sens	photop_sens
TS	HC	650	400	640	2.5	2.5
	VC1	715	440	640	2.5	2.5
	VC2	650	400	704	2.5	2.5
	VC3	715	440	704	2.5	2.5
NG	HC	590	460	590	2.4	2.5
	VC1	649	506	590	2.4	2.5
	VC2	590	460	650	2.4	2.5
	VC3	649	506	650	2.4	2.5
HM	HC	610	500	600	2.4	2.5
	VC1	671	550	600	2.4	2.5
	VC2	610	500	660	2.4	2.5
	VC3	671	550	660	2.4	2.5
ZMD	HC	505	460	620	2.3	2.5
	VC1	555	506	620	2.3	2.5
	VC2	505	460	682	2.3	2.5
	VC3	555	506	682	2.3	2.5

*Note that vern_sens is sensitivity to vernalization; photop_sens is sensitivity to photoperiod; tt_end_of_juvenile is thermal time required from end of juvenile to floral initiation (°Cd); tt_floral_initiation is thermal time required from floral initiation to flowering (°Cd); and startgf_to_mat is thermal time required from flowering to grain filling (°Cd).*

To derive the optimum sowing date for wheat in the NCP under SSP245 and SSP585 scenarios, the yield performance for four investigated stations was evaluated with different sowing dates ranging from 20 days before historical sowing date (Table 1) to 30 days after historical sowing date with an interval of 5 days in the two future periods (2040S and 2080S).

## Results

### Projected Future Climate Change

Changes in mean daily solar radiation and temperature (i.e., T_max_ and T_min_) and total annual precipitation for the two future periods (2040S and 2080S) relative to the baseline period based on the downscaled data from the 20 GCMs under SSP245 and SSP585 scenarios were shown in Supplementary Figure S2. For T_max_ and T_min_, four investigated stations showed significantly increasing trends in all the GCMs (Supplementary Figures S2B,C). For solar radiation, there were increasing trends at all the investigated stations for the two future periods under both SSP245 and SSP585 scenarios (Supplementary Figure S2A). Moreover, the increase in mean daily radiation during 2080S at four stations under SSP245 scenario was greater than that under SSP585 scenario (Supplementary Figure S2A). As for precipitation, the average change projected by 20 GCMs showed that precipitation increased at all the stations for two future periods under SSP245 and SSP585 scenarios (Supplementary Figure S2D). Moreover, precipitation variability projected by 20 GCMs was large.

**Figure 2:**
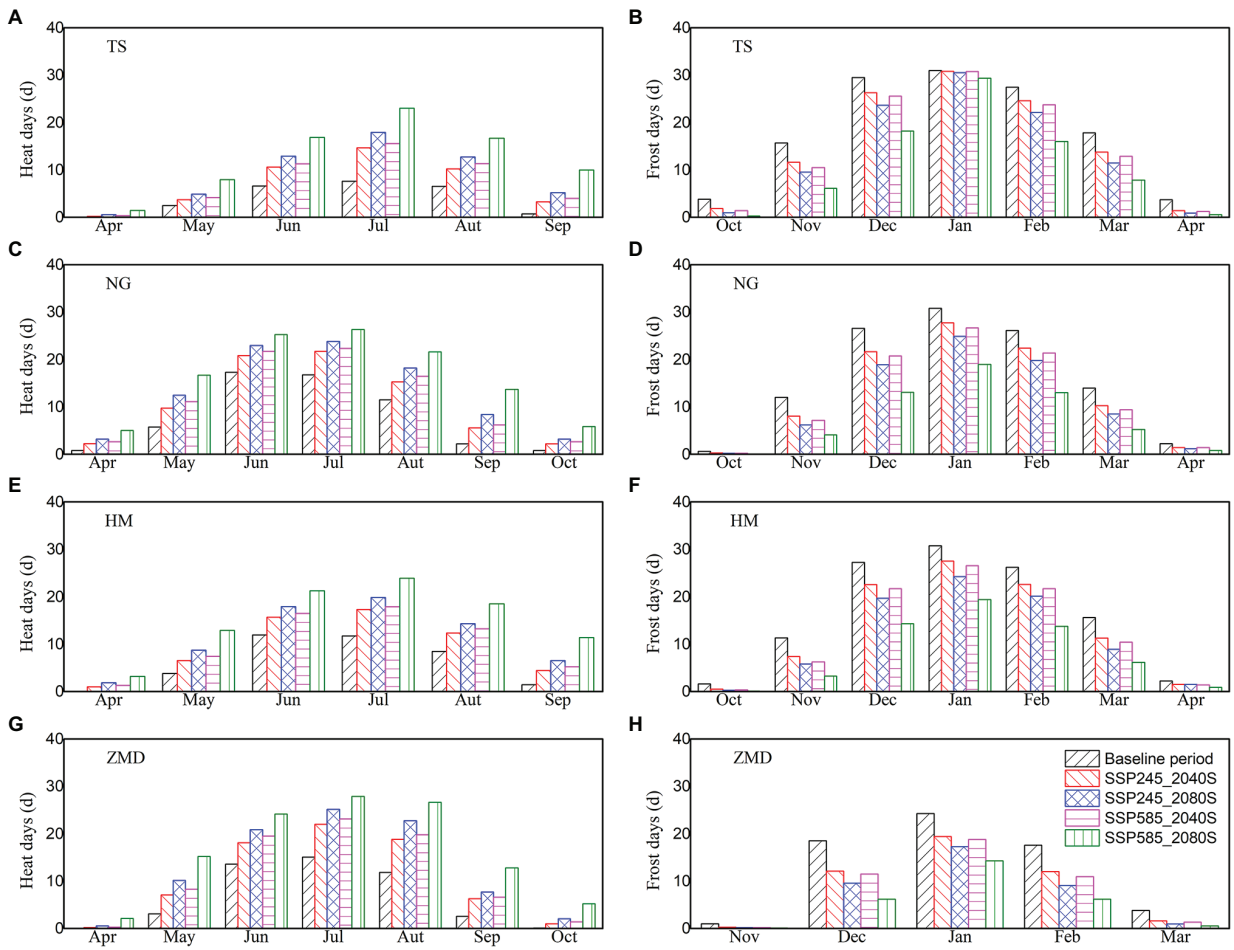
Monthly heat **(A,C,E,G)** and frost **(B,D,F,H)** days for baseline period and two future periods under SSP245 and SSP585 scenarios.

Monthly heat (the number of days with T_max_ ≥ 32°C) and frost (the number of days with T_min_ < 0°C) days for baseline period and two future periods under SSP245 and SSP585 scenarios are shown in Figure 2. Along with climate warming, heat days in each month increased significantly at all the stations under future climate scenarios. Heat days were mainly concentrated from May to September (Figures 2A,C,E,G). In the baseline period, wheat bloomed in April at ZMD station and in May at other stations. Across the stations, the average number of heat days in May projected by 20 GCMs increased by 1.2–4.0 and 1.7–5.3 days under SSP245 and SSP585 scenarios during 2040S, respectively, and increased by 2.4–7.1 and 5.4–12.2 days under SSP245 and SSP585 scenarios during 2080S, respectively (Figures 2A,C,E,G). Heat days in April projected by 20 GCMs increased slightly. Frost days in each month decreased significantly at all the stations under future climate scenarios (Figures 2B,D,F,H). Frost days were mainly concentrated from December to February of the following year at ZMD station and concentrated from November to March of the following year at other stations. In the baseline period, wheat jointed in March at ZMD station and in April at other stations. There will be almost no frost days in March at ZMD station and no frost days in April at other stations under the future climate scenario (Figures 2B,D,F,H).

### Shift in Wheat Phenology Under Future Climate Scenarios

**Figure 3:**
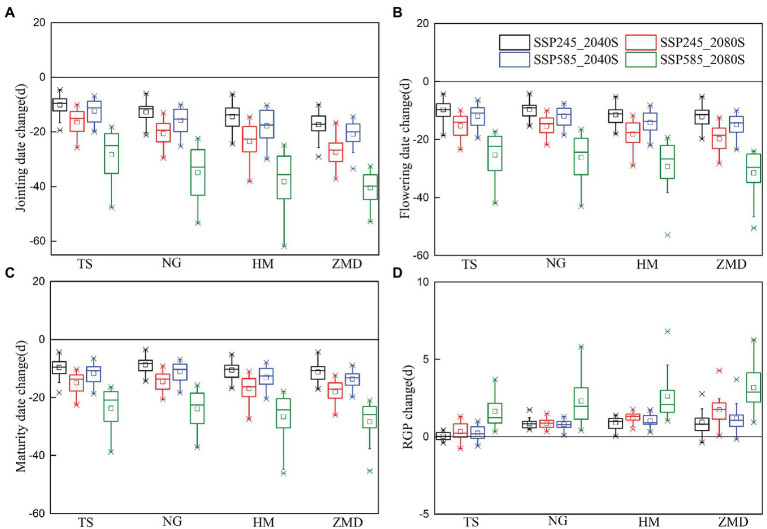
Changes in jointing **(A)**, flowering **(B)**, and maturity **(C)** dates and the reproductive growth phase (RGP), **(D)** in the 2040S and 2080S under SSP245 and SSP585 scenarios relative to the baseline (1981–2010).

Generally, warming climate could accelerate crop development rate and consequently reduce crop growth duration. In this study, the simulation results showed that there was a significant advancing trend in wheat phenology (e.g., jointing date, flowering date, and maturity date) under future climate scenarios (Figure 3). The advance in the days of wheat phenology during 2080S was greater than that during 2040S. Moreover, changes in wheat phenology under SSP585 scenario were greater than those under SSP245 scenario (Figure 3). As the sowing date remained unchanged for the two future periods, the advance in flowering and maturity dates significantly shortened the VGP (duration from sowing to flowering) and the whole growth phase (WGP, duration from sowing to maturity) of wheat. However, the RGP (duration from flowering to maturity) of wheat was prolonged by 0.2–1.3 and 0.4–1.7 days across the stations under SSP245 and SSP585 scenarios during 2040S, respectively, and prolonged by 0.5–2.5 and 2.1–5.3 days under two scenarios during 2080S, respectively (Figure 3D). The main reason for the extension of the RGP was that the early trend in maturity date was less than that in the flowering date.

### Impacts of Climate Change and Extreme Temperature on Wheat Yield

**Figure 4:**
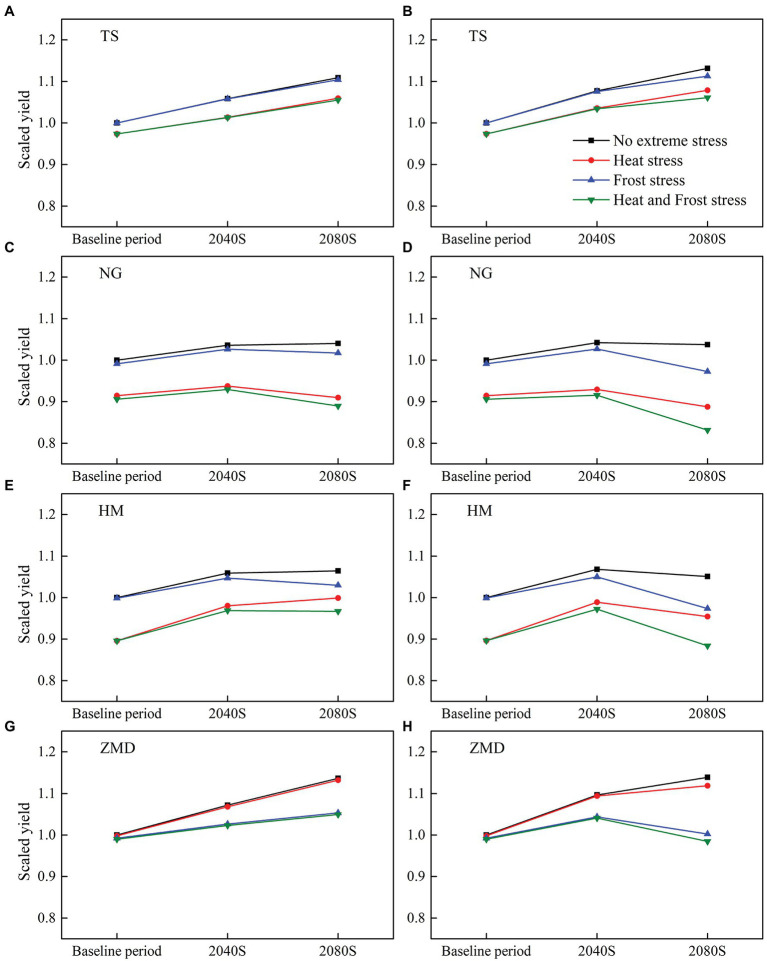
Simulated multi-Global Climate Model (GCM) ensemble mean yield for the baseline and two future periods (2040S and 2080S) under SSP245 **(A,C,E,G)** and SSP585 **(B,D,F,H)** without and with the effect of extreme temperature stress (i.e., heat and frost stress). All the yields have been normalized by historical yield without the effect of extreme temperature events.

Winter wheat yields for the baseline and two future periods were investigated with and without considering the effect of extreme temperature (Figure 4). Without considering the effect of extreme temperature, wheat yield showed an increasing trend at all the stations under future climate scenarios. The yield increase was largest at ZMD station, increasing by averages of 7.2, 13.6, 9.7, and 13.9% across 20 GCMs under SSP245_2040S, SSP245_2080S, SSP585_2040S, and SSP585_2080S, respectively (Figures 4G,H).

**Figure 5:**
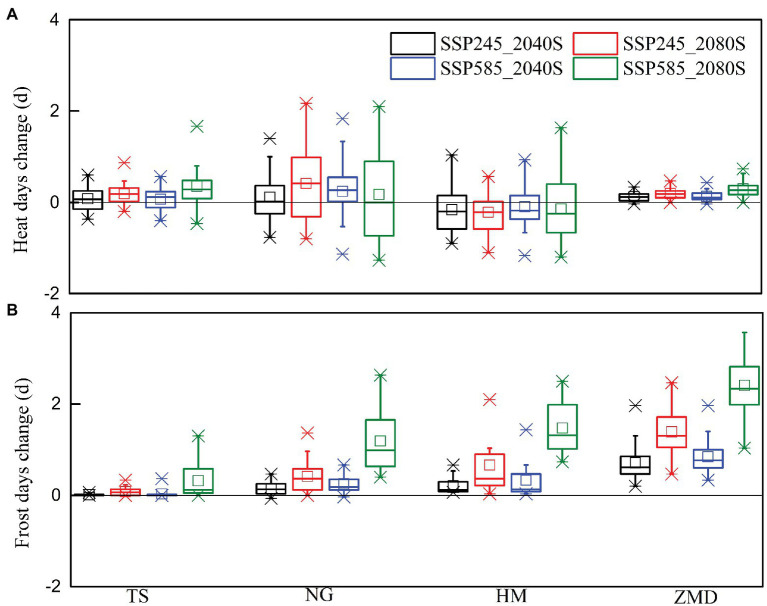
Changes in heat **(A)** and frost days **(B)** during the temperature sensitive stage of wheat in the 2040S and 2080S under SSP245 and SSP585 scenarios compared to the baseline period (1981–2010).

**Figure 6:**
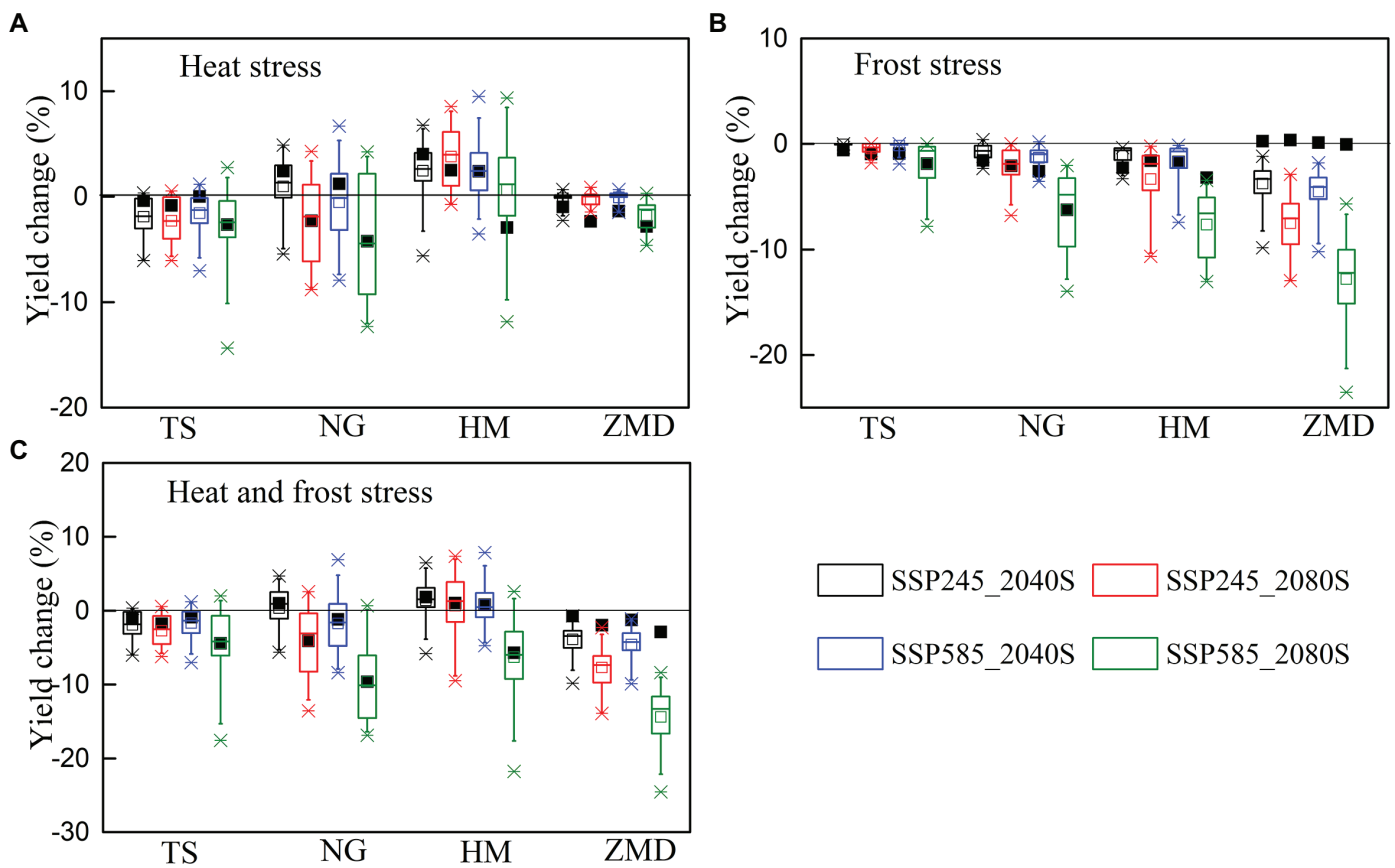
Changes in impacts of heat **(A)**, frost **(B)**, heat and frost stress **(C)** on wheat yield in the 2040S and 2080S under SSP245 and SSP585 scenarios compared to the baseline period (1981–2010). Black rectangles represent multi-model mean values from simulations with optimum adaptation.

The effects of extreme temperature events, including heat stress and frost stress, on yield have been accounted for in this study. In the baseline period, yield change caused by heat stress at NG and HM stations was large, but that at the other two stations was slight (Figure 4). The main reason was that the number of heat days during the high temperature sensitive stage of wheat at NG and HM stations was more than that at other stations in the baseline period (Table 1; Figure 2). Under future climate scenarios, heat days during flowering and grain filling period had a slight decrease at HM station but a slight increase at the other three stations (Figure 5A). As a result, wheat yield loss caused by heat stress at HM station decreased by 0.6–3.7% under future climate scenarios, but that at the other stations increased slightly (Figure 6A). With the combined effects of climate change and heat stress, wheat yield had an increasing trend under future climate scenarios at all the stations except for NG station (Figure 4).

Frost stress had no significant effect on yield at all the stations for the baseline period (Figure 4). However, due to the advancement of wheat phenology in the future climate scenarios, frost days from jointing to grain filling had a significant increase at all the stations except for TS station, especially for SSP585_2080S (Figure 5B). Wheat yield loss caused by frost stress increased by 3.7–12.8% at ZMD, but that at other stations increased slightly (except for NG and HM under SSP585_2080S; Figure 6B). Moreover, wheat yield loss caused by frost stress during 2080S was greater than that during 2040S. Wheat yield loss caused by frost stress under SSP585 scenario was larger than that under SSP245 scenario (Figure 6B). With the combined effects of climate change and frost stress, wheat yield had an increasing trend at TS station under future climate scenarios. However, wheat yield increased in 2040S and decreased in 2080S at NG, HM, and ZMD (except for 2040S) stations (Figure 4). Moreover, with the combined effects of climate change and heat and frost stress, wheat yield change was similar to that only considering the effect of climate change and frost stress under the future climate scenarios (Figure 4). Overall, wheat yield loss caused by heat and frost stress increased by 3.9–14.4% at ZMD, but that had no significant change under all climate scenarios except for SSP585_2080S at other stations (Figure 6C).

### Responses of Wheat Yield to Cultivar and Sowing Date Adjustment

**Figure 7:**
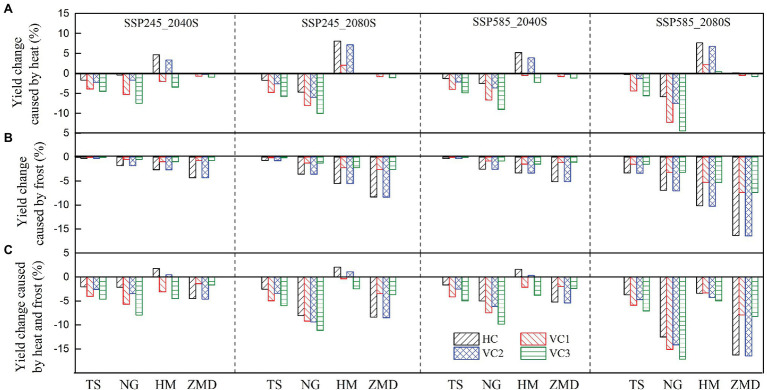
The relative change of impact of heat **(A)**, frost **(B)**, heat and frost stress **(C)** for different cultivars on wheat yield in the 2040S and 2080S under SSP245 and SSP585 scenarios relative to historical cultivar in the baseline (1981–2010).

Without considering the effects of extreme climate stress (e.g., heat and frost stress), wheat yield under VC2 cultivar showed the best performance at all the stations in two future periods under both SSP245 and SSP585 scenarios (Supplementary Figure S3A). Yield loss caused by heat stress using HC cultivar was smaller than using other virtual cultivars at all stations under future climate scenarios, and yield loss using VC3 cultivar was largest (Figure 7A). Moreover, cultivars changes had no significant effect on yield loss caused by heat stress at ZMD station. Yield loss caused by frost stress with VC1 and VC3 cultivars was less than with HC and VC2 at all stations for future climate scenarios (Figure 7B). Yield loss caused by heat and frost stress with VC1 cultivar was least at ZMD station, but yield loss with HC cultivar was least at other stations (Figure 7C). With the effect of extreme stress, wheat yield with VC1 cultivar showed the best performance at ZMD station for future climate scenarios, but wheat yield with HC cultivar showed the best performance at other stations (Supplementary Figure S3B). According to the simulated results, the optimum cultivar was HC cultivar at TS, NG, and HM stations, and the optimum cultivar was VC1 cultivar at ZMD station.

**Figure 8:**
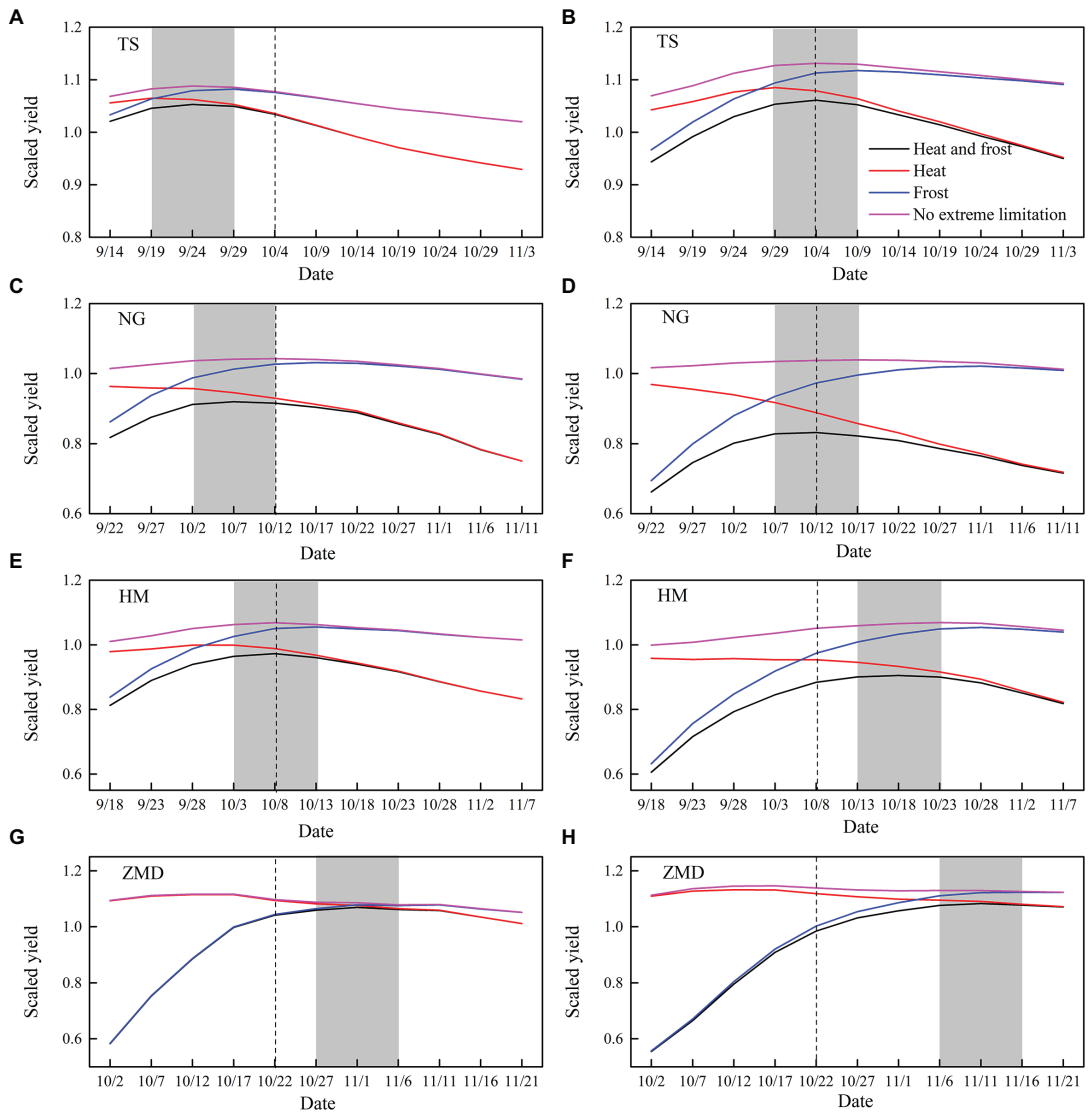
Yield performance considering heat and frost stress in the different sowing dates during the 2040S **(A,C,E,G)** and 2080S **(B,D,F,H)** under SSP245 scenario. The black dotted line is the historical sowing date. The gray rectangle is the optimum sowing window. All the yields have been normalized by historical yield without the effect of extreme temperature events.

With the combined effect of heat and frost stress, responses of wheat yield to sowing dates in two future periods under SSP245 and SSP585 scenarios were shown in Figure 8 and Supplementary Figure S4, respectively. With the advance of wheat sowing date from historical sowing date, the negative effect of heat stress on yield decreased at all stations except ZMD station under future climate scenarios. However, the negative effect of frost stress on yield significantly increased at all the stations under future climate scenarios (Figure 8; Supplementary Figure S4). However, with the delay of wheat sowing date from historical sowing date, the negative effect of heat stress on yield significantly increased at all the stations except for ZMD station under future climate scenarios, but the negative effect of frost stress on yield significantly decreased at all the stations except for TS station in 2040S (Figure 8; Supplementary Figure S4).

Under the combined effect of heat and frost stress, the fluctuation of yield change caused by shift in sowing date reached −42.8–2.0, −47.7–5.3, −45.8–2.8, and −43.0–9.8% under the four future climate scenarios, that is, SSP245_2040S, SSP245_2080S, SSP585_2040S, and SSP585_2080S, respectively, relative to the historical sowing date (Figure 8; Supplementary Figure S4). The optimum sowing window was to advance historical sowing date by 5–15 days for 2040S under SSP245 and SSP585 scenario at TS station, respectively. The optimum sowing window was to advance historical sowing date by 0–10 days at NG and HM stations during 2040S under the two climate scenarios, but the optimum sowing window was to delay historical sowing date by 5–15 days at ZMD station (Figure 8; Supplementary Figure S4). Moreover, for all the stations during 2080S under the two climate scenarios, the optimum sowing window was 5–10 days later than that during 2040S (Figure 8; Supplementary Figure S4).

The relative changes of the impact of extreme temperature stress on wheat yield with optimum adaptation of sowing date and cultivar selection in the 2040S and 2080S under SSP245 and SSP585 scenarios relative to the baseline period were shown in Figure 6. The simulation results showed that there is a trade-off between the effects of heat and frost stress on yield with optimum adaptation. The optimum adaptation decreased the effect of heat stress on yield at TS and NG, but increased the effect of frost stress on yield (Figures 6A,B). The optimum adaptation significantly decreased the yield loss caused by the combined effect of heat and frost stress by 3.2–11.5% at ZMD under the future climate scenarios, but slightly reduced the yield loss at the other stations (Figure 6C).

## Discussion

Changes and variability of climate factors, such as temperature, solar radiation, precipitation, and [CO_2_] during crop growing season, could strongly influence crop phenology and productivity (Lin et al., 2005; Tao et al., 2014; Zhang et al., 2016). Based on statistically downscaled daily climate data from 20 GCMs in CMIP6, we found that radiation, temperature (T_min_ and T_max_), and precipitation have an increasing trend under future climate scenarios. Overall, the trends were consistent with the result of Xiao et al. (2018b) using 28 GCMs from CMIP5. Generally, warming climate could accelerate crop growth and development rate and thereby advance crop phenological stages (Xiao et al., 2015; Hu et al., 2017). The length of VGP and WGP of wheat significantly shortened for the two future periods under SSP245 and SSP585 scenarios. The large decrease of crop VGP could cause insufficient biomass accumulation and lower leaf area index (LAI) in the early growth stage and has a negative impact on the accumulation of grain yield in the later growth stage (Juknys et al., 2017). However, the length of RGP was prolonged under future climate scenarios, which could provide the possibility of prolonging grain filling period and increasing grain yield (Wang and Yin, 2012). To some extent, increasing precipitation could improve soil moisture conditions during crop growing season (Rosenzweig et al., 2004). The increase in precipitation in the future could reduce irrigation water consumption and effectively alleviate the problems caused by overexploitation of groundwater in the area. However, Xiao et al. (2020) found that some areas in the northern NCP still had groundwater over-pumping in the future based on 33 GCMs from CMIP5. Irrigation is still an important guarantee for high yield of wheat in rainfed areas of the NCP. Solar radiation is an important climatic factor which influences photosynthesis rate and biomass accumulation of crop (Sheehy et al., 2006; Zhang et al., 2010). Increased radiation under future climate scenarios could benefit wheat production (Xiao et al., 2020). However, the shortening of growth period could result in less radiation interception (Xiao et al., 2018b). The elevated atmospheric CO_2_ concentration under future climate scenarios has positive effects on crop yield, especially for the C3 plant wheat (Chen et al., 2018; Xiao et al., 2018b).

Without considering the effect of extreme climate, climate change increased winter wheat yield under both SSP245 and SSP585 scenarios in the NCP. However, due to warming climate, extreme climate events are anticipated to increase, which are likely to produce negative impacts on crop production (Porter and Semenov, 2005). When the crop phenology changes in the future are not considered, Bai et al. (2020) noted that the frequency and intensity of heat extremes during wheat growing season were projected to increase over the 21st century for SSP245 and SSP585 scenarios, but those of cold extremes will decrease. In this study, the results showed that heat days around flowering had no significant change or a slight decrease for wheat under SSP245 and SSP585 scenarios. Frost days from jointing to grain filling had a slight increase under future climate scenarios but significantly increased for SSP585_2080S scenario. The main reason for the differences was that our study considered the changes of wheat phenology caused by warming climate. Therefore, the actual changes of heat and frost stress are not only attributed to temperature change but also the variation of crop phenology (Gu et al., 2008). Increase in temperature accelerates wheat development and advances the temperature sensitive stage, which could reduce the risk of exposure to heat stress but increase the risk of frost (Zheng et al., 2012). Related studies also indicated that although rising temperature reduced spring frost significantly in most of wheat growing region, actual risk of spring frost during jointing to flowering had not decreased as expected and frost-related yield loss had an increasing trend (Zheng et al., 2015; Xiao et al., 2018b). Our simulation results also showed that wheat yield loss caused by frost stress significantly increased for 2080S under SSP585 scenario, especially for ZMD station. This is because the temperature sensitive stage of wheat was earlier at ZMD than that at the other three stations under future climate scenarios (the jointing date was advanced from March to February; Figure 3). Although frost days in each month decreased significantly due to the increase of temperature, the number of frost day in February under future climate scenarios were still significantly higher than those in March for the baseline period (Figure 2H).

The projected yield changes under future climate scenarios not only presented possible risks to crop production but also suggested potential opportunities for agricultural development. The negative impact of warming climate on crop generally resulted from the decrease in growth duration and the increase of extreme events (Chen et al., 2018). Although increasing temperature accelerated crop development rate, the variation of phenology was not completely consistent with the temperature variation (He et al., 2015; Li et al., 2016). Shift of crop phenology can be affected not only by climatic factors but also by cultivar changes and agronomic management (Tao et al., 2012; Wang et al., 2013). An optimum sowing window can reduce risks of frost and/or heat stress events during temperature sensitive stage (Zheng et al., 2012; Bell et al., 2015). In this study, we found that the fluctuation of yield change caused by shift in sowing dates under future climate scenarios was very large. Proper shift of sowing window could alleviate the negative effect of frost and heat stress events and maintain or even improve wheat yields. The optimum sowing window of wheat was different in different regions, which was controlled by climatic conditions and cultivar characteristics (Bai and Tao, 2017). Moreover, not all cultivars respond similarly to climate change, and cultivars renewal is the main mean of adaptation to climate change (Lobell et al., 2011). Several studies indicated that long-season cultivar could compensate for phenology acceleration induced by warming climate and stabilize wheat growth duration (Liu et al., 2010; Tao et al., 2012). Late sowing dates or using long-season cultivars could postpone the frost sensitive stage and decrease frost risk, but increase the risk of exposing wheat to more heat stress around flowering (Zheng et al., 2012). Therefore, the selection of adaptation measures needs to balance the risks associated with frost, heat, and other abiotic stresses (Barlow et al., 2015). In this study, we found late sowing and longer VGP cultivars could significantly compensate for the negative impact of extreme temperature on yields at ZMD in the south of NCP under future climate scenarios, but shift in sowing dates and growth period length of cultivars only slightly compensated for the negative impact at TS, NG, and HM in the north and central NCP. The results showed that changing the key growth period by management measures cannot effectively alleviate the combined effects of extreme temperature for wheat in the north and central NCP, and improving the tolerance to high temperature or low temperature of cultivars may be an effective measure to alleviate the negative effects of extreme temperature.

There are some uncertainties in future climate projections due to the differences between different climate models and scenarios. We used a multiple model ensemble method to address the uncertainties from climate models and scenarios. To assess the impacts of extreme temperature on wheat production, we integrated yield reduction multipliers for heat and frost stress events during temperature sensitive stage into APSIM-wheat model based on relevant research reports. Due to limited data for model evaluation, our modeling results might over- or underestimate the magnitude of yield losses resulted from heat and frost stress. Nonetheless, capturing heat and frost losses to grain yield in some way could provide guidance for developing adaptation strategies to reduce climate risks (Bell et al., 2015). Further improvement of the definitions and physiological basis of this approach would enhance the accuracy of these predictions, but this is out of the scope of this study. Moreover, in the APSIM-wheat model, the increased photosynthesis due to elevated atmospheric [CO_2_] was reported mainly from controlled, semi-controlled, and open-field experiments (Reyenga et al., 1999; Kimball et al., 2002). Therefore, the crop model might overestimate the positive effects of elevated atmospheric [CO_2_] (Asseng et al., 2004). In order to more accurately evaluate the impact of climate change on crop production, the quality of the crop model and climate projection should be further elaborated.

## Conclusion

Based on statistically downscaled data from 20 GCMs in CMIP6, we evaluated the potential changes in wheat phenology and yield across winter wheat cropping regions in the NCP using the APSIM-wheat model. Results showed that warming climate accelerated wheat development and significantly advanced the temperature sensitive stage. Without any adaptation methods under future climate scenarios, the risk of exposure to heat stress around flowering had no significant change or a slight decrease, but frost risk in spring season increased. Extreme temperature stress would have negative impacts on wheat production. However, agricultural climate resources, such as light, thermal, and CO_2_ fertilization effects, could partly compensate for the yield decrease or even contribute to the yield increase. Moreover, we found that late sowing and longer VGP cultivar could significantly compensate for the negative impact of extreme temperature on wheat yields in the south of NCP under future climate scenarios. However, selecting heat resistant cultivars in the north NCP and both heat and frost resistant cultivars in the central NCP may be a more effective way to alleviate the negative effect of extreme temperature on wheat yields. Our analysis highlighted that not only heat risk should be concerned under climate warming, but also frost risk should not be ignored. Therefore, exploring agricultural management measures to balance the risks associated with frost, heat, and other abiotic stresses should be the priority to ensure wheat production in the NCP.

## Data Availability Statement

The raw data supporting the conclusions of this article will be made available by the authors, without undue reservation.

## Author Contributions

HB and DX carried out the study design and wrote the original draft. HB carried out the data collection and model simulation. BW, DL, and JT also gave critical revision of the manuscript. All authors contributed to the article and approved the submitted version.

## Funding

This study was supported by the Natural Science Foundation of China (41901128) and the Natural Science Foundation of Hebei Province (D2018302012).

## Conflict of Interest

The authors declare that the research was conducted in the absence of any commercial or financial relationships that could be construed as a potential conflict of interest.

## Publisher’s Note

All claims expressed in this article are solely those of the authors and do not necessarily represent those of their affiliated organizations, or those of the publisher, the editors and the reviewers. Any product that may be evaluated in this article, or claim that may be made by its manufacturer, is not guaranteed or endorsed by the publisher.

## Footnotes

^1^ https://esgf-node.llnl.gov/search/cmip6/
