# Supplementary material for: Simulation of Wheat Response to Future Climate Change Based on Coupled Model Inter-Comparison Project Phase 6 Multi-Model Ensemble Projections in the North China Plain
Source: Front Plant Sci. 2022 Feb 3;13:829580. doi: 10.3389/fpls.2022.829580 (PMC8850353; doi:10.3389/fpls.2022.829580)
Supplement: Supplementary file 1 [file Data_Sheet_1.docx]

Supplementary materials for

**Simulation of wheat response to future climate change based on CMIP6 multi-model ensemble projections in the North China Plain**

Huizi Bai^1^, Dengpan Xiao^1, 2^*, Bin Wang^2^, De Li Liu^2,3^, Jianzhao Tang^1^

^1^ Engineering Technology Research Center, Geographic Information Development and Application of Hebei, Institute of Geographical Sciences, Hebei Academy of Sciences, Shijiazhuang 050011, China

^2^ NSW Department of Primary Industries, Wagga Wagga Agricultural Institute, Wagga Wagga, New South Wales 2650, Australia

^3^ Climate Change Research Centre and ARC Centre of Excellence for Climate Extremes, University of New South Wales, Sydney, New South Wales 2052, Australia

* Correspondence: Dengpan Xiao, Email: [xiaodp@sjziam.ac.cn](mailto:xiaodp@sjziam.ac.cn)





**Fig. S1 Validation plots of the APSIM-wheat model simulation on jointing date, flowering date, maturity date and yield for winter wheat at the study stations.**





**Fig. S2 Changes in solar radiation (Rad, a), maximum temperature (Tmax, b), minimum temperature (Tmin, c), and precipitation (Prec, d) for the 2040S (2031–2060) and 2080S (2071–2100) under SSP245 and SSP585 scenarios relative to the baseline (1981–2010).**





**Fig. S3 Yield changes of different cultivar without (a) and with (b) extreme temperature stress in the 2040S and 2080S under SSP245 and SSP585 scenarios. The yields have been normalized by historical yield without the effect of extreme temperature events.**





**Fig. S4 Yield performance considering heat and frost stress in the different sowing dates during the 2040S (a, c, e, g) and 2080S (b, d, f, h) under SSP585 scenario. The black dotted line is the historical sowing date. The grey rectangle is the optimum sowing window. All the yields have been normalized by historical yield without the effect of extreme temperature events.**
